# Preoperative Weight Trends in Adolescents Undergoing Metabolic and Bariatric Surgery

**DOI:** 10.1002/oby.70136

**Published:** 2026-01-29

**Authors:** Sarah B. Ogle, Emily H. Meneses, Alexander M. Kaizer, Jaime M. Moore, James E. Mitchell, Marc P. Michalsky, Thomas Inge

**Affiliations:** ^1^ Pediatrix Medical Group Orem Utah USA; ^2^ Department of Pediatrics Section of Nutrition, University of Colorado School of Medicine, Children's Hospital Colorado Aurora Colorado USA; ^3^ Department of Biostatistics and Informatics, Colorado School of Public Health University of Colorado Anschutz Medical Campus Aurora Colorado USA; ^4^ Sanford Biobehavioral Research Institute and School of Medicine and Health Sciences University of North Dakota Fargo North Dakota USA; ^5^ Department of Pediatric Surgery Nationwide Children's Hospital and the Ohio State University College of Medicine Columbus Ohio USA; ^6^ Department of Surgery, Ann & Robert H. Lurie Children's Hospital of Chicago Northwestern University Feinberg School of Medicine Chicago Illinois USA

**Keywords:** adolescent, bariatric, preoperative weight

## Abstract

**Objective:**

Preoperative weight changes, predictors of weight changes, and subsequent implications on postoperative BMI reduction in adolescents preparing for bariatric surgery (MBS) have not been well described.

**Methods:**

Teen–Longitudinal Assessment of Bariatric Surgery (Teen‐LABS) consortium (prospective, observational MBS study at five centers from 2007 to 2011) participants who completed the preoperative phase within 3–9 months of initial visit were included in this analysis (*n* = 123). Participants were categorized into preoperative weight groups: > 1% loss, stable, or > 1% gain. Demographic, anthropometric, socioeconomic, medical, and behavioral data were analyzed. Postoperative percent BMI loss at 1, 5, and 8 years by weight group was compared.

**Results:**

Preoperatively, 50% of participants lost weight, 20% remained stable, and 30% gained weight. The mean percent weight change by group was −4.2% (standard deviation [SD] 2.9%), +0.02% (SD 0.6%), and +5.2% (SD 5.3%), respectively. Eight‐year postoperative BMI change was −21% (lost) and −26% (stable), compared to −15% among those who gained weight preoperatively (*p* = 0.11). No differences in preoperative weight‐related behaviors were observed between groups.

**Conclusions:**

Most adolescents preparing for MBS maintained ±5% of their baseline weight. No statistically significant differences in postoperative BMI loss or factors predicting preoperative weight change were identified.

**Trial Registration:**

ClinicalTrials.gov identifier: NCT00474318

## 1 Introduction

Prior to undergoing metabolic and bariatric surgery (MBS), adolescents are typically enrolled in multidisciplinary programs that assess their medical and psychological candidacy for weight loss surgery. These programs also aim to optimize lifestyle behaviors, manage medical comorbidities, provide education, and address other clinical and psychosocial needs. While considerable variability in preoperative weight changes has been described in the adult MBS literature, with weight stability or weight gain being common [1], such changes and subsequent postoperative implications during the preoperative period have not yet been well described in adolescents.

Adult studies have not consistently demonstrated a benefit of preoperative weight loss on postoperative complications or weight outcomes. The typical 6‐month requirement for weight management imposed by insurance companies tends to delay surgery, increase attrition, and may lead to progression of obesity‐related diseases [2, 3, 4, 5]. In 2016, the American Society for Metabolic and Bariatric Surgery (ASMBS) updated its position statement on insurance‐mandated preoperative weight management and questioned the benefit to patients [6]. The American Academy of Pediatrics similarly advocates for increased access to MBS, including reducing insurance barriers, eliminating unsubstantiated exclusion criteria, and minimizing delays in surgical treatment due to denials of insurance authorization for indicated procedures [7, 8].

Preoperative predictors of postoperative weight loss have been elusive in the adult MBS population [9]. Weight changes in the preoperative phase of adolescent MBS candidates have not been critically analyzed and reported. This study analyzes the preoperative weight changes of adolescents preparing for MBS, evaluates associations between preoperative weight changes and select clinical variables, and examines whether preoperative weight changes predict postoperative weight loss.

## 2 Methods

### 2.1 Participants

Teen–Longitudinal Assessment of Bariatric Surgery (Teen‐LABS) study research methods, diagnostic criteria, and data collection have previously been described [10, 11]. The Teen‐LABS observational study is registered at ClinicalTrials.gov (NCT00474318). Briefly, between 2007 and 2012, 242 adolescents (≤ 19 years of age) who were undergoing MBS at five centers in the United States were enrolled. Michalsky et al. described the programmatic and institutional characteristics of the five MBS centers that constitute the Teen‐LABS research consortium [12]. The study protocol, assent/consent forms, and data and safety monitoring plans were approved by the institutional review boards of each institution and by the independent data and safety monitoring board prior to study initiation.

For all participants enrolled into Teen‐LABS, preprogram weight at the time of initial referral to the MBS program was obtained from their medical records (preprogram weight) and was prospectively abstracted into the study database. For this analysis we included only those 123 participants whose time of referral to the MBS program was between 90 and 270 days prior to MBS. This inclusion criterion was used as this time period reflects the typical duration of preoperative preparation seen at these clinical sites. Baseline preoperative data, including demographic, anthropometric, and comorbidity data, were also prospectively collected per protocol within 30 days prior to the surgical procedure (baseline weight).

Postoperatively, research visits were conducted at 6 months, 12 months, and then annually. This analysis evaluated data collected up to 8 years post MBS. During this time frame, 95% of those enrolled remained as active study participants, completing 86% of all protocol‐mandated postoperative research visits. The pre‐ and postoperative clinical care of participants was directed by the clinicians at each center where enrollment occurred and was not dictated by the research protocol. However, clinical care generally followed national guideline recommendations, and similarities across these programs were previously described by Michalsky et al. [12].

### 2.2 Data Collection

Demographic, anthropometric, socioeconomic, medical, and self‐reported behavioral data were collected at baseline and at annual study visits. Behavioral metrics addressing nutrition, physical activity, weight self‐monitoring, utilization of support services, presence of disordered eating, sleep hygiene, and presence of substance abuse were collected using standardized surveys. Variables selected for this analysis were modeled after a preoperative weight analysis by the adult Longitudinal Assessment of Bariatric Surgery consortium [13]. These data were analyzed to identify variables associated with weight loss, stability, or gain during the preoperative period. Table S1 summarizes preoperative adolescent weight control behaviors, and primary caregiver characteristics analyzed are shown in Table S2.

### 2.3 Weight Trends

Teen‐LABS defined preprogram weight as the weight at the time of referral of patients to their multidisciplinary MBS program. Baseline (within 30 days prior to operation) and postoperative weight and height were collected and used to calculate body mass index (BMI). Weight and BMI measurements at visits associated with pregnancy were excluded. Participants undergoing vertical sleeve gastrectomy (VSG), Roux‐en‐Y gastric bypass (RYGB), and laparoscopic adjustable banding (LAGB) were included in the analysis since the preoperative interventions were not different among individuals who would subsequently undergo these various procedures. Participants were categorized arbitrarily into three preoperative weight change groups by comparing preprogram weight and baseline weight prior to surgery: (1) preoperative weight loss group, defined as loss of > 1% of their preprogram weight during the preoperative phase, (2) weight stable group, defined as those who maintained within ±1%, and (3) weight gain group defined as gaining > 1% of their preprogram weight during the preoperative phase.

### 2.4 Statistical Methods

Continuous measures are presented as mean (standard deviation [SD]) and median (1st quartile, 3rd quartile). Categorical measures are presented as count (percent). Shapiro‐Wilks tests were used to determine normality of continuous measures and ANOVA or Kruskal–Wallis tests, where appropriate, were used to make comparisons across the three preoperative weight trend groups. For categorical variables, chi‐square or Fisher's exact tests, where appropriate, were used to compare the distribution of responses between groups. We did not correct for multiple comparisons in this exploratory analysis. All analyses were conducted in RStudio version 1.2.1335 with R version 4.0.2 (Vienna, Austria).

## 3 Results

### 3.1 Participant and Caregiver Demographics

Table 1 describes participant demographic and surgery type by preoperative weight change group. There were no statistical differences among the examined demographic variables or surgery type by weight group. Table 2 describes primary caregiver characteristics including education level, income, insurance status, and history of MBS. There were no significant differences in preoperative weight trends by primary caregiver characteristics.

**TABLE 1:** Demographics and surgery type by preoperative weight trend group.

	Loss (*N* = 62)	Stable (*N* = 24)	Gain (*N* = 37)	*p*
Age (years)				
Mean (SD)	16.4 (1.63)	16.7 (1.8)	16.6 (1.8)	0.66
Median [Min, Max]	16.5 [13, 20]	17 [13, 19]	17.0 [13.0, 19.0]	
Sex				
Male	13 (21%)	4 (16.7%)	11 (29.7%)	0.44
Female	49 (79%)	20 (83.3%)	26 (70.3%)	
Race				
White	47 (75.8%)	18 (75.0%)	28 (75.7%)	1
Black	10 (16.1%)	4 (16.7%)	7 (18.9%)	
American Indian or Alaska Native	1 (1.6%)	0 (0%)	0 (0%)	
More than one race	4 (6.5%)	2 (8.3%)	2 (5.4%)	
Surgery				
Roux‐en‐Y gastric bypass	40 (64.5%)	18 (75%)	25 (67.6%)	0.83
Sleeve gastrectomy	20 (32.3%)	6 (25%)	10 (27%)	
Laparoscopic adjustable gastric band	2 (3.2%)	0 (0%)	2 (5.4%)	

*Note*: Age, sex, race, and surgery type were similar between preoperative weight groups.

**TABLE 2:** Primary caregiver characteristics by preoperative weight trend group.

	Loss (*N* = 62)	Stable (*N* = 24)	Gain (*N* = 37)	*p*
Highest education level completed by primary caregiver?				
Less than high school	2 (3.2%)	2 (8.3%)	0 (0%)	0.87
Some high school (grades 9–12, no diploma or GED)	5 (8.1%)	1 (4.2%)	4 (10.8%)	
Some home‐schooling (grades 9–12, no diploma or GED)	1 (1.6%)	0 (0%)	0 (0%)	
General Equivalency Degree (GED)	5 (8.1%)	2 (8.3%)	3 (8.1%)	
Graduated from high school	12 (19.4%)	4 (16.7%)	6 (16.2%)	
1 to 2 years of college, no degree	11 (17.7%)	3 (12.5%)	9 (24.3%)	
3 or more years of college, no degree	6 (9.7%)	0 (0%)	4 (10.8%)	
Graduated from a 2‐year college or associate's	11 (17.7%)	3 (12.5%)	4 (10.8%)	
Graduated from a college/university and bachelor's	5 (8.1%)	4 (16.7%)	4 (10.8%)	
Some graduate school courses	2 (3.2%)	1 (4.2%)	1 (2.7%)	
Master's degree	1 (1.6%)	1 (4.2%)	1 (2.7%)	
Professional degree	0 (0%)	1 (4.2%)	0 (0%)	
Missing	1 (1.6%)	2 (8.3%)	1 (2.7%)	
Which of the categories below represents the primary caregiver's annual household income before taxes?				
Less than $5000	1 (1.6%)	3 (12.5%)	1 (2.7%)	0.52
$5000–$14,999	11 (17.7%)	3 (12.5%)	5 (13.5%)	
$15,000–$24,999	14 (22.6%)	1 (4.2%)	8 (21.6%)	
$25,000–$49,999	10 (16.1%)	6 (25.0%)	9 (24.3%)	
$50,000–$74,999	11 (17.7%)	3 (12.5%)	4 (10.8%)	
$75,000–$99,999	5 (8.1%)	2 (8.3%)	5 (13.5%)	
$100,000–$199,999	6 (9.7%)	3 (12.5%)	4 (10.8%)	
$200,000 or more	2 (3.2%)	0 (0%)	0 (0%)	
Missing	2 (3.2%)	3 (12.5%)	1 (2.7%)	
Does the primary caregiver have medical insurance?				
No	6 (9.7%)	6 (25%)	8 (21.6%)	0.09
Yes	55 (88.7%)	16 (66.7%)	28 (75.7%)	
Missing	1 (1.6%)	2 (8.3%)	1 (2.7%)	
Does the primary caregiver have a history of bariatric surgery?				
No	45 (72.6%)	15 (62.5%)	26 (70.3%)	0.62
Yes	13 (21.0%)	7 (29.2%)	7 (18.9%)	
Missing	4 (6.5%)	2 (8.3%)	4 (10.8%)	

*Note*: Socioeconomic and primary caregiver bariatric status were similar between preoperative weight groups.

### 3.2 Weight Trends

**FIGURE 1:**
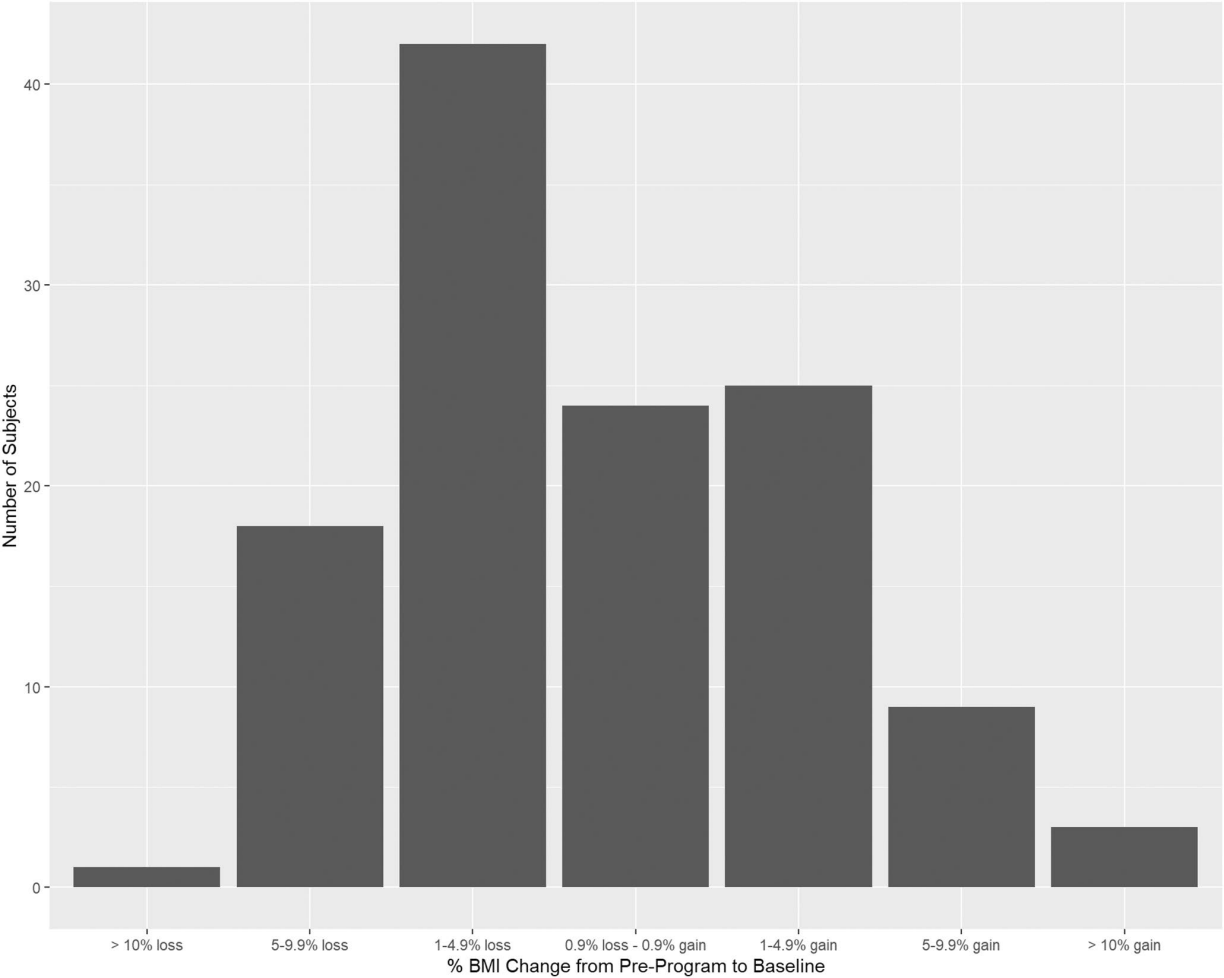
Distribution of preoperative BMI change. BMI remained within ±5% during the preoperative phase for most participants.

One hundred and twenty‐three (51%) of all enrollees had their preprogram and baseline weights documented between 90 and 270 days prior to their date of MBS. Within this analysis cohort, Group 1 (weight loss) consisted of 62 (50%) participants whose baseline (immediately preoperative) mean weight was 4.2% (SD 2.9%) lower than their preprogram weight. Group 2 (weight stable) consisted of 24 (20%) individuals whose mean baseline weight was +0.02% (SD 0.6%) higher than their preprogram weight, and Group 3 (weight gain) consisted of 37 (30%) participants who gained on average 5.2% (SD 5.3%) above their preprogram weight (Table 3). Figure 1 illustrates the overall preoperative BMI trends distribution with most participants (> 90%) staying within ±5% of their baseline BMI prior to MBS.

**TABLE 3:** Weight and BMI change over time by preoperative weight group.

	Loss (*N* = 62)	Stable (*N* = 24)	Gain (*N* = 37)	*p*
Percent weight change: preprogram to baseline				
Mean (SD)	−4.18 (2.9)	0.02 (0.6)	5.18 (5.3)	< 0.001
Median (Q1, Q3)	−3.6 (−5.2, −2.2)	−0.13 (−0.5, 0.5)	3.39 (2.4, 6.4)	
Time from preprogram to baseline (days)				
Mean (SD)	176 (45.6)	191 (37.1)	194 (48.7)	0.14
Median (Q1, Q3)	169 (147, 206)	195 (169, 209)	199 (154, 225)	
Preprogram BMI (kg/m^2^)				
Mean (SD)	53.4 (9.3)	49.8 (9)	50.2 (9)	0.03*
Median (Q1, Q3)	51.8 (47.0, 57.9)	47.0 (43.7, 53)	47.1 (43.0, 57.1)	
Missing	1 (1.6%)	0 (0%)	0 (0%)	
BMI at baseline (kg/m^2^)				
Mean (SD)	51.3 (8.2)	49.7 (8.5)	52.2 (9.2)	0.41
Median (Q1, Q3)	50.2 (45.2, 55.3)	47.3 (43.6, 52.4)	48.0 (45.1, 58.5)	
BMI at 1 year (kg/m^2^)				
Mean (SD)	35.3 (8.1)	35.3 (9.1)	38 (9.2)	0.32
Median (Q1, Q3)	34.5 (29.6, 39)	33.9 (29.5, 37.2)	37.1 (31.2, 41.7)	
Missing	9 (14.5%)	5 (20.8%)	4 (10.8%)	
BMI at 5 years (kg/m^2^)				
Mean (SD)	39.4 (13)	33.8 (8.5)	42.8 (12.1)	0.03
Median (Q1, Q3)	38.1 (30.6, 45.9)	31.1 (27.4, 38.4)	41.9 (33.1, 51.3)	
Missing	18 (29%)	7 (29.2%)	6 (16.2%)	
BMI at 8 years (kg/m^2^)				
Mean (SD)	41.3 (13.1)	37.5 (10.2)	44.5 (13.3)	0.26
Median (Q1, Q3)	41.2 (34.6, 45.2)	37.2 (26.7, 43.2)	40.9 (33.8, 51.4)	
Missing	21 (33.9%)	6 (25%)	13 (35.1%)	
Percent change in BMI: preprogram to baseline				
Mean (SD)	−3.99 (3.2)	−0.036 (2.8)	4.09 (5.6)	< 0.001
Median (Q1, Q3)	−3.9 (−5.5, −1.8)	0.06 (−1.1, 1.4)	2.6 (1.5, 5.1)	
Missing	1 (1.6%)	0 (0%)	0 (0%)	
Percent change in BMI: baseline to 1 year				
Mean (SD)	−31 (9)	−30.1 (10)	−28.7 (11)	0.57
Median (Q1, Q3)	−30.5 (−37.2, −26.6)	−27.8 (−36.9, −23.8)	−31.5 (−37.1, −18.8)	
Missing	9 (14.5%)	5 (20.8%)	4 (10.8%)	
Percent change in BMI: baseline to 5 years				
Mean (SD)	−23.4 (15.6)	−30.0 (15.0)	−18.6 (17.9)	0.07
Median (Q1, Q3)	−24.3 (−34.4, −9.8)	−33.5 (−44.2, −16.5)	−13.4 (−33, −7.6)	
Missing	18 (29%)	7 (29.2%)	6 (16.2%)	
Percent change in BMI: baseline to 8 years				
Mean (SD)	−20.8 (16.3)	−25.8 (14.1)	−14.8 (19.5)	0.11
Median (Q1, Q3)	−17.2 (−28.2, −9.5)	−26.4 (−34.1, −13.7)	−15.3 (−24.8, −1.1)	
Missing	21 (33.9%)	6 (25%)	13 (35.1%)	

*Note*: Percent weight change and preprogram BMI were significantly different between preoperative weight groups. Asterisk identifies *p* values reaching significance. BMI 5 years after surgery (*p* = 0.03) was lowest in patients who were weight stable prior to surgery.

**FIGURE 2:**
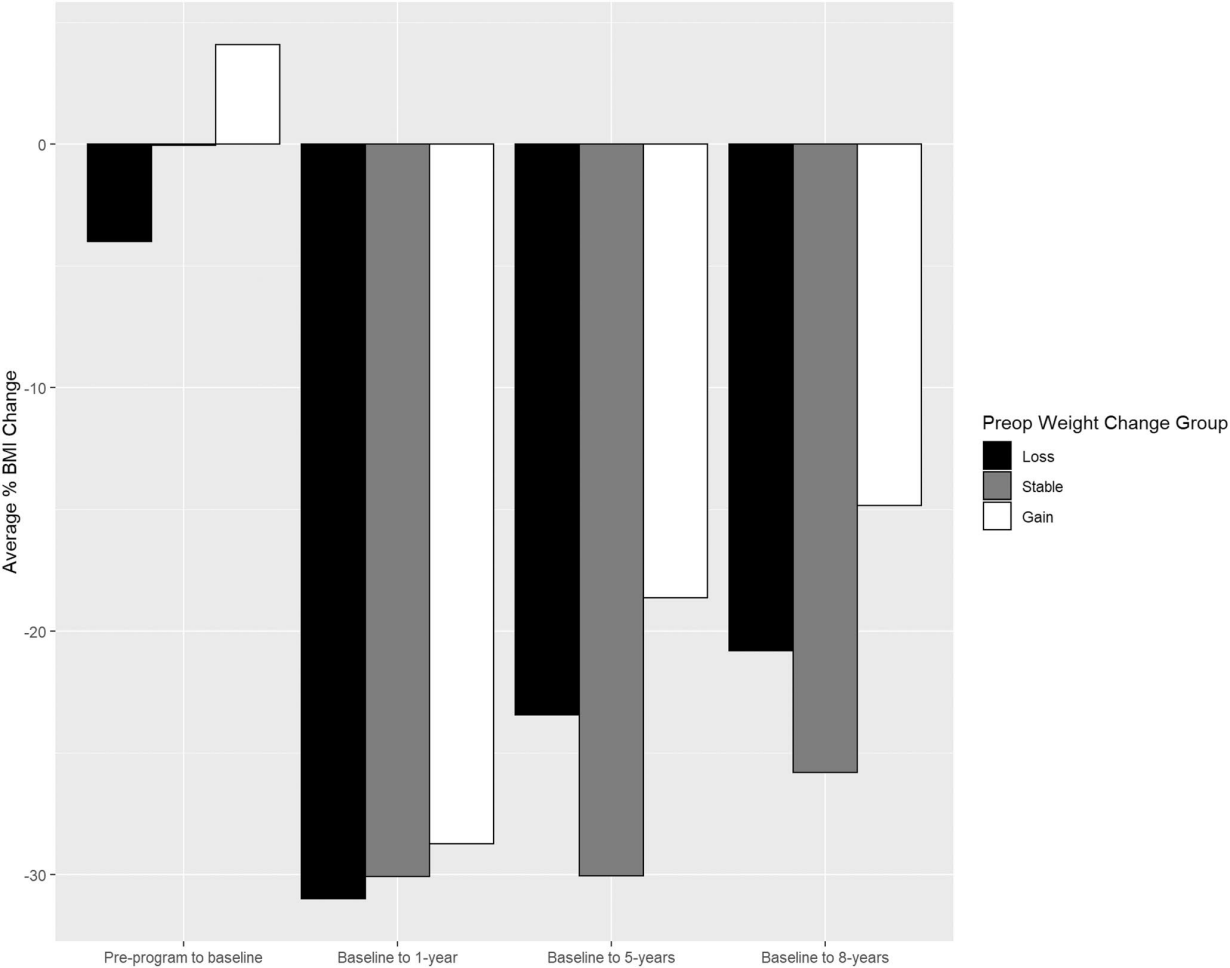
Preoperative weight trend group and postoperative percent BMI change. Percent BMI change at 1, 5, and 8 years after surgery was not significantly different between preoperative weight groups.

Table 3 describes preoperative and postoperative weight and BMI metrics by weight change group. There were no significant differences in baseline (*p* = 0.41), 1‐year (*p* = 0.32), or 8‐year (*p* = 0.26) absolute BMI by preoperative weight change group. BMI 5 years after surgery was statistically different (*p* = 0.03), with weight stable participants having the lowest mean BMI (33.8 kg/m^2^, SD 8.5), followed by weight loss (39.4 kg/m^2^, SD 13) and weight gain (42.8 kg/m^2^, SD 12.1) groups. There were no significant differences in percent BMI loss at 1, 5, and 8 years after MBS (*p* = 0.57, *p* = 0.07, and *p* = 0.11, respectively). Notably, while statistical significance was not observed, a pattern at 5 and 8 years after MBS, with participants in the weight stable group experiencing a greater percent BMI reduction (−30%, SD 15% and −25.8%, SD 14%) compared to those with weight loss (−23.4%, SD 16% and −20.8%, SD 16%) and weight gain (−18.6%, SD 18% and −14.8%, SD 20%). Figure 2 shows the trends of percent BMI loss after MBS for each preoperative weight change group over time.

### 3.3 Weight Control Behaviors

The relationship between weight‐related behaviors and preoperative weight change group was examined (Tables S1 and S2). Table S1 lists the behavior characteristics that were investigated in this analysis. Table S2 describes behavior characteristics by group. There were few characteristics that were found to have significant differences among weight groups.

Participants who were advised or required to lose weight prior to undergoing MBS (*p* = 0.02) and participants who were advised or required to start a special diet prior to MBS (*p* = 0.03) lost significantly more weight in the preoperative period. However, diet type (*p* = 0.3) and dietary adherence (*p* = 0.4) were not significantly different between groups. Difficulty falling asleep in the 3 months prior to starting the MBS program was also significantly different (*p* = 0.04).

## 4 Discussion

This Teen‐LABS subanalysis defined three preoperative weight change groups in adolescents awaiting MBS, with most adolescents staying within ±5% of their baseline BMI. Participants who maintained weight prior to MBS experienced nonsignificant greater weight loss, particularly at 5 and 8 years after surgery. Analysis of possible associated factors revealed that participants who were asked to lose weight or to start a special diet preoperatively were more likely to be in the weight loss or stability group, though there were no statistical significances between diet type or adherence between weight groups. Additionally, difficulty falling asleep within 3 months of initiating MBS program responses reached significance (*p* = 0.04); however, no clinically meaningful pattern was identified to guide care.

In one of very few studies evaluating weight changes during the preoperative phase in adolescents, Fennig et al. examined the relationship between preoperative weight loss and postoperative weight loss at 6 months in 48 adolescents at a single center undergoing VSG in Israel [14]. Notably, their preoperative program included weekly sessions over 3 months. They found a quadratic relationship between preoperative BMI reduction and 6‐month postoperative BMI change, where moderate preoperative BMI reduction (2.4‐point BMI reduction) was associated with greater BMI reduction at 6 months post MBS compared to either extreme (minimal or very significant preoperative BMI reduction). The authors hypothesized that this might reflect adoption of desired lifestyle targets in anticipation of MBS. Our study complements these findings by providing data from a larger, multicenter cohort and at later time points of 1, 5, and 8 years postoperatively.

The modest amount of preoperative weight loss demonstrated in our cohort is similar to prior studies in adults [15, 16]. There has been advocacy in the adult MBS population to remove insurance‐mandated preoperative medical weight management prior to MBS approval, since it has shown little postoperative benefit [6, 9, 13, 17]. The current study suggests that achieving weight stability prior to MBS in adolescents may predict a postoperative BMI benefit. Our study was, however, not designed to evaluate the ideal duration of preoperative weight stability or optimal magnitude of preoperative weight change which may be beneficial to maximize postoperative weight loss.

The lack of association between specific weight‐related behaviors and preoperative weight loss changes suggests that an individualized approach to the length of preoperative preparation may be more beneficial than an arbitrary number of months or visits imposed by a payer. Prior adult studies demonstrated self‐weighing as a predictor of pre‐MBS weight loss [18], a finding that we did not reproduce in this sample of adolescents. Neither socioeconomic status nor caregiver history of MBS was statistically associated with preoperative weight trends in this study. The influence of socioeconomic status and preoperative weight loss has not been thoroughly explored in the literature. However, food insecurity and education level could contribute to a patient's weight trajectory [19, 20]. Emerging evidence suggests that a caregiver's history of MBS has shown either a neutral or positive impact on offspring and other family member weight trends after MBS; however, additional studies are needed to determine whether this effect is primarily driven by biological/genetic or environmental factors [21, 22].

Certainly, core preoperative education, nutrition, and medical optimization are necessary to safely prepare adolescents and their caregivers for postoperative expectations but should be balanced against unnecessary delays that could lead to progression of complications of obesity or higher attrition [23]. Future studies could explore prescribed durations and degrees of preoperative medical weight loss versus an individual patient readiness approach [24]. In the adult MBS population, prolonged durations and specific medical weight loss goals in the preoperative phase have not been shown to predict postoperative outcomes or prevent postoperative complications [5, 25, 26, 27]. Preparing adolescents for MBS may also present unique challenges as, compared to adults, medical decision‐making, their home environment, and exposure to food are heavily influenced by other people in the household. Additionally, variable baseline maturity and increasing autonomy as adolescents transition to young adulthood present challenges that are different from the adult MBS population when preparing for long‐term lifestyle changes and weight maintenance.

This study was not designed to evaluate the influence of myriad baseline factors on postoperative weight trajectory. Prior attempts to identify predictive preoperative factors on postoperative outcomes in adolescents undergoing MBS have been limited. An analysis of the Follow‐up of Adolescent Bariatric Surgery at 5 Plus years MBS cohort did not demonstrate any differences in postoperative lifestyle or weight behaviors relating to postoperative weight loss [28].

This study also indirectly affirms, similar to many previous studies, that despite engagement in lifestyle and medical management on an approximate monthly or bimonthly basis during the preoperative phase, most adolescents with severe obesity were able to achieve only modest weight change. Importantly, the 5‐ and 8‐year postoperative BMI outcomes described in this study could suggest that a reasonable expectation for adolescents in the preoperative period is to achieve bariatric program educational goals and weight stability.

4.1

This was a secondary analysis of data collected primarily for other purposes, and thus limitations include the observational nature of the study and lack of a design that specifically focused on questions related to preoperative weight loss. Preoperative weight management and preparation for MBS care generally followed national guidelines; however, differences across the five participating institutions may have influenced preoperative weight management results [29]. While we did not account for change in growth velocity, the Teen‐Lab cohort has previously demonstrated stable height from baseline to postoperative time points and the preoperative phase analysis notes similar changes in percent weight change and percent BMI change suggesting stable height [11]. Additionally, since most participants were advised to actively participate in weight management strategies in a short‐term period prior to undergoing MBS, the preoperative weight loss observed in many of the participants should not be considered generalizable to all adolescents with severe obesity who are not seeking surgery. It should be noted that self‐reported preoperative nutritional guidance reported by participants may not accurately reflect the actual nutritional guidance delivered by study or clinical staff during the preoperative phase [12]. Future studies could further investigate participant understanding of nutritional guidance, preoperative and postoperative BMI trajectory, and overall MBS readiness.

Strengths of the study included the relatively large sample size, the long‐term BMI outcome at 8 years, and the structured, prospective design of the parent study. While the study participant demographics are generalizable to MBS patients, this distribution also reflects the underutilization of MBS in males and minorities with obesity [30, 31]. Finally, for most of the variables used, completeness of data collection was good to excellent.

## 5 Conclusion

Most adolescents awaiting MBS maintained within ±5% of their baseline BMI. Though not statistically significant, participants who maintained their baseline weight prior to undergoing MBS tended to have greater percent BMI loss at 5 and 8 years after surgery. Preoperative weight trajectory was not predictable based on weight related behaviors.

## Funding

Funding for the Teen‐LABS consortium included the following: National Institute of Diabetes and Digestive and Kidney Diseases (NIDDK) grants: UM1DK072493 (University of Colorado) and UM1DK095710 (University of Cincinnati). The study was also supported by grants UL1 TR000077‐04 (Cincinnati Children's Hospital Medical Center), UL1RR025755 (Nationwide Children's Hospital), M01‐RR00188 (Texas Children's Hospital/Baylor College of Medicine), UL1 RR024153 and UL1TR000005 (University of Pittsburgh), and UL1 TR000165 (University of Alabama, Birmingham). Sponsors did not participate in the work presented.

## Conflicts of Interest

Thomas Inge has received consulting fees from Teleflex, Medtronic, and Eli Lilly Inc. and royalties from Wolters Kluwer, none of which was related to the content of this manuscript. Marc P. Michalsky received honoraria from Intuitive Surgical Inc. and has been an independent owner of Intuitive Surgical Inc. stock, both unrelated to the content of this manuscript. Emily H. Meneses receives grant funding from Boehringer Ingelheim for work unrelated to this study. All other authors declare no conflicts of interest.

## Supporting information


**Table S1:** General description of weight related behavioral characteristics analyzed.
**Table S2:** Analysis of preoperative behaviors by preoperative weight trend group. Few characteristics were found to be statistically significant. Asterisk identifies *p* values reaching significance. Participants who were advised/required to lose weight (*p* = 0.02) or start a special diet (*p* = 0.03) lost weight prior to surgery. Sleep hygiene was statistically different between weight groups (*p* = 0.04).

## Data Availability

Deidentified individual participant data (including data dictionaries) are available via the NIDDK Central Repository (https://repository.niddk.nih.gov/home).
