# Supplementary material for: Preoperative Weight Trends in Adolescents Undergoing Metabolic and Bariatric Surgery
Source: Obesity (Silver Spring). 2026 Jan 29;34(3):710–8. doi: 10.1002/oby.70136 (PMC12933217; doi:10.1002/oby.70136)
Supplement: Supplementary file 1 — Table S1: General description of weight related behavioral characteristics analyzed. Table S2: Analysis of preoperative behaviors by preoperative weight trend group. Few characteristics were found to be statistically significant. Asterisk identifies p values reaching significance. Participants who were advised/required to lose weight (p = 0.02) or start a special diet (p = 0.03) lost weight prior to surgery. Sleep hygiene was statistically different between weight groups (p = 0.04). [file OBY-34-710-s001.docx]

Supplemental

**Table S1. Summary of behavioral characteristics**

| **Behaviors** |  |  |  |  |
| --- | --- | --- | --- | --- |
| Required to lose weight  Required to start a special diet  Self-weighing  Meal history, snacking  Fast food consumption  Eating out  Tobacco, alcohol, substance abuse  Eat after dinner  Recent weight gain/loss  Sedentary time  Sleep hygiene  History of seeing mental health provider  History of seeing dietitian  History of seeing exercise specialist  History of disordered eating and associated behaviors  History of weight loss medication use  History of shift work | | | | |

**Supplemental**

**Table S2: Relationship between preoperative behaviors and preoperative weight trend group**

|  | **Loss (N=62)** | **Stable (N=24)** | **Gain (N=37)** | **P-value** |
| --- | --- | --- | --- | --- |
| **Were you advised or required by your doctor or other health care provider to lose weight prior to your obesity surgery?** |  |  |  |  |
| No | 10 (16.7%) | 7 (29.2%) | 15 (42.9%) | 0.02* |
| Yes | 50 (83.3%) | 17 (70.8%) | 20 (57.1%) |  |
| Missing | 2 (3.2%) | 0 (0%) | 2 (5.4%) |  |
| **How much weight were you advised or required to lose? (lbs)** |  |  |  |  |
| Mean (SD) | 41.8 (61) | 48 (47.6) | 20 (10) | 0.94 |
| Median (Q1, Q3) | 20 (15, 40) | 20 (10, 100) | 20 (15, 25) |  |
| Missing | 53 (85.5%) | 19 (79.2%) | 34 (91.9%) |  |
| **Were you advised or required by your doctor or other health care provider to start a special diet prior to your obesity surgery?** |  |  |  |  |
| No | 12 (20%) | 8 (33.3%) | 16 (45.7%) | 0.03* |
| Yes | 48 (80%) | 16 (66.7%) | 19 (54.3%) |  |
| Missing | 2 (3.2%) | 0 (0%) | 2 (5.4%) |  |
| **What kind of special diet?** |  |  |  |  |
| High protein/low carbohydrate | 8 (17%) | 3 (20%) | 1 (5.9%) | 0.28 |
| One or more of the above diets | 15 (31.9%) | 3 (20%) | 9 (52.9%) |  |
| Other | 10 (21.3%) | 3 (20%) | 4 (23.5%) |  |
| Very low calorie | 14 (29.8%) | 6 (40%) | 2 (11.8%) |  |
| Ground of pureed foods | 0 (0%) | 0 (0%) | 1 (5.9%) |  |
| Missing | 15 (24.2%) | 9 (37.5%) | 20 (54.1%) |  |
| **Did you follow the special diet?** |  |  |  |  |
| No | 0 (0%) | 0 (0%) | 1 (5.3%) | 0.45 |
| Rarely | 0 (0%) | 0 (0%) | 0 (0%) |  |
| Occasionally | 3 (6.3%) | 3 (18.8%) | 1 (5.3%) |  |
| Usually | 24 (50%) | 6 (37.5%) | 8 (42.1%) |  |
| Always | 21 (43.8%) | 7 (43.8%) | 9 (47.4%) |  |
| Missing | 14 (22.6%) | 8 (33.3%) | 18 (48.6%) |  |
| **Do you weigh yourself at least weekly preoperatively?** |  |  |  |  |
| No | 36 (97.3%) | 18 (100%) | 23 (100%) | 1 |
| Yes | 1 (2.7%) | 0 (0%) | 0 (0%) |  |
| Missing | 25 (40.3%) | 6 (25%) | 14 (37.8%) |  |
| **For weight control, have you ever: seen a counselor/mental health professional?** |  |  |  |  |
| No | 45 (75%) | 18 (75%) | 24 (68.6%) | 0.77 |
| Yes | 15 (25%) | 6 (25%) | 11 (31.4%) |  |
| Missing | 2 (3.2%) | 0 (0%) | 2 (5.4%) |  |
| **How many times in the 6 months prior to coming to this program have you seen a counselor/mental health professional?** |  |  |  |  |
| 0 | 2 (13.3%) | 0 (0%) | 0 (0%) | 0.05 |
| 1-5 times | 8 (53.3%) | 1 (16.7%) | 7 (63.6%) |  |
| 6-10 times | 4 (26.7%) | 3 (50%) | 0 (0%) |  |
| 11-20 times | 0 (0%) | 1 (16.7%) | 1 (9.1%) |  |
| More than 20 times | 1 (6.67%) | 1 (16.7%) | 3 (27.3%) |  |
| Missing | 47 (75.8%) | 18 (75%) | 26 (70.3%) |  |
| **For weight control, have you ever seen a nutritionist/dietitian?** |  |  |  |  |
| No | 22 (37.3%) | 6 (25%) | 15 (42.9%) | 0.39 |
| Yes | 37 (62.7%) | 18 (75%) | 20 (57.1%) |  |
| Missing | 3 (4.8%) | 0 (0%) | 2 (5.4%) |  |
| **How many times in the 6 months prior to coming to this program have seen a nutritionist/dietitian?** |  |  |  |  |
| 0 | 6 (16.2%) | 2 (11.1%) | 2 (10%) | 0.93 |
| 1-5 times | 21 (56.8%) | 8 (44.4%) | 10 (50%) |  |
| 6-10 times | 6 (16.2%) | 6 (33.3%) | 5 (25%) |  |
| 11-20 times | 2 (5.4%) | 1 (5.6%) | 2 (10%) |  |
| More than 20 times | 2 (5.4%) | 1 (5.6%) | 1 (5%) |  |
| Missing | 25 (40.3%) | 6 (25%) | 17 (45.9%) |  |
| **For weight control, have you ever seen a personal trainer or exercise specialist?** |  |  |  |  |
| No | 44 (73.3%) | 13 (56.5%) | 26 (74.3%) | 0.27 |
| Yes | 16 (26.7%) | 10 (43.5%) | 9 (25.7%) |  |
| Missing | 2 (3.2%) | 1 (4.2%) | 2 (5.4%) |  |
| **How many times in the 6 months prior to coming to this program have seen a personal trainer or exercise specialist?** |  |  |  |  |
| 0 | 4 (25%) | 1 (10%) | 1 (11.1%) | 0.43 |
| 1-5 times | 1 (6.25%) | 3 (30%) | 3 (33.3%) |  |
| 6-10 times | 3 (18.8%) | 3 (30%) | 0 (0%) |  |
| 11-20 times | 3 (18.8%) | 2 (20%) | 3 (33.3%) |  |
| More than 20 times | 5 (31.3%) | 1 (10%) | 2 (22.2%) |  |
| Missing | 46 (74.2%) | 14 (58.3%) | 28 (75.7%) |  |
| **How many days out of the 7-day week do you eat breakfast?** |  |  |  |  |
| Mean (SD) | 5.5 (2) | 5.2 (2.1) | 5.23 (2.2) | 0.78 |
| Median (Q1, Q3) | 6.5 (4, 7) | 6.(3.8, 7) | 6 (4, 7) |  |
| Missing | 2 (3.2%) | 0 (0%) | 2 (5.4%) |  |
| **How many days out of the 7-day week do you eat lunch/brunch?** |  |  |  |  |
| Mean (SD) | 6.6 (1.2) | 6.5 (1.2) | 6.5 (0.95) | 0.81 |
| Median (Q1, Q3) | 7 (7, 7.) | 7 (7, 7) | 7 (7, 7) |  |
| Missing | 2 (3.2%) | 0 (0%) | 2 (5.4%) |  |
| **How many days out of the 7-day week do you eat dinner?** |  |  |  |  |
| Mean (SD) | 6.8 (0.56) | 6.8 (0.57) | 6.9 (0.37) | 0.77 |
| Median (Q1, Q3) | 7 (7, 7) | 7 (7, 7) | 7 (7, 7) |  |
| Missing | 2 (3.2%) | 0 (0%) | 2 (5.4%) |  |
| **Counting all meals and any snacks you may have, how many times a day do you eat?** |  |  |  |  |
| Mean (SD) | 4.6 (1.7) | 4.7 (1) | 4.2 (1.3) | 0.39 |
| Median (Q1, Q3) | 4 (3.8, 5) | 4.5 (4, 5) | 4.(3, 5) |  |
| Missing | 2 (3.2%) | 0 (0%) | 4 (10.8%) |  |
| **How many days a week do you eat out at fast food restaurants for breakfast?** |  |  |  |  |
| Mean (SD) | 0.32 (0.75) | 0.42 (1.1) | 0.2 (0.47) | 0.83 |
| Median (Q1, Q3) | 0 (0, 0) | 0 (0, 0) | 0 (0, 0) |  |
| Missing | 3 (4.8%) | 0 (0%) | 2 (5.4%) |  |
| **How many days a week do you eat out at fast food restaurants for lunch?** |  |  |  |  |
| Mean (SD) | 0.73 (1.1) | 0.79 (1.1) | 0. (1.4) | 0.88 |
| Median (Q1, Q3) | 0 (0, 1) | 0 (0, 1.6) | 0 (0, 1) |  |
| Missing | 2 (3.2%) | 0 (0%) | 2 (5.4%) |  |
| **How many days a week do you eat out at fast food restaurants for dinner?** |  |  |  |  |
| Mean (SD) | 1.1 (1.3) | 1.2 (1.2) | 1.4 (1.5) | 0.84 |
| Median (Q1, Q3) | 1 (0, 1.3) | 1 (0, 2) | 1 (0, 2) |  |
| Missing | 2 (3.2%) | 0 (0%) | 2 (5.4%) |  |
| **How many days a week do you eat out at other types of restaurants for breakfast?** |  |  |  |  |
| Mean (SD) | 0.17(0.5) | 0.13 (0.34) | 0.09 (0.38) | 0.65 |
| Median (Q1, Q3) | 0 (0, 0) | 0 (0, 0) | 0 (0, 0) |  |
| Missing | 3 (4.8%) | 0 (0%) | 4 (10.8%) |  |
| **How many days a week do you eat out at other types of restaurants for lunch?** |  |  |  |  |
| Mean (SD) | 0.39 (0.81) | 0.42 (0.72) | 0.3 (0.7) | 0.65 |
| Median (Q1, Q3) | 0 (0, 0.5) | 0 (0, 1) | 0 (0, 0) |  |
| Missing | 3 (4.8%) | 0 (0%) | 4 (10.8%) |  |
| **How many days a week do you eat out at other types of restaurants for dinner?** |  |  |  |  |
| Mean (SD) | 1 (1.3) | 0.92 (0.78) | 0.76 (1.1) | 0.43 |
| Median (Q1, Q3) | 1 (0, 1) | 1 (0, 1.3) | 0 (0, 1) |  |
| Missing | 3 (4.8%) | 0 (0%) | 4 (10.8%) |  |
| **During the 6 months prior to coming to this program, have you had times when you eat continuously during the day or parts of the day without planning what and how much you would eat?** |  |  |  |  |
| No | 32 (53.3%) | 13 (54.2%) | 20 (57.1%) | 0.94 |
| Yes | 28 (46.7%) | 11 (45.8%) | 15 (42.9%) |  |
| Missing | 2 (3.2%) | 0 (0%) | 2 (5.4%) |  |
| **Did you experience a loss of control, that is, you felt like you could not control your eating?** |  |  |  |  |
| No | 11 (39.3%) | 5 (45.5%) | 5 (33.3%) | 0.82 |
| Yes | 17 (60.7%) | 6 (54.5%) | 10 (66.7%) |  |
| Missing | 34 (54.8%) | 13 (54.2%) | 22 (59.5%) |  |
| **During the 6 months prior to coming to this program, did you ever eat within any two-hour period what most people would regard as an unusually large amount of food?** |  |  |  |  |
| No | 36 (60%) | 14 (58.3%) | 24 (68.6%) | 0.64 |
| Yes | 24 (40%) | 10 (41.7%) | 11 (31.4%) |  |
| Missing | 2 (3.2%) | 0 (0%) | 2 (5.4%) |  |
| **During the 3 months prior to coming to this program, did you ever take more than twice the recommended dose of diuretics (water pills) in order to avoid gaining weight after binge eating?** |  |  |  |  |
| No | 10 (100%) | 4 (100%) | 8 (100%) |  |
| Yes | 0 (0%) | 0 (0%) | 0 (0%) |  |
| Missing | 52 (83.9%) | 20 (83.3%) | 29 (78.4%) |  |
| **During the 3 months prior to coming to this program, did you ever fast (not eating anything at all for at least 24 hours) in order to avoid gaining weight after binge eating?** |  |  |  |  |
| No | 10 (100%) | 4 (100%) | 6 (75%) | 0.15 |
| Yes | 0 (0%) | 0 (0%) | 2 (25%) |  |
| Missing | 52 (83.9%) | 20 (83.3%) | 29 (78.4%) |  |
| **During the 3 months prior to coming to this program, did you ever take twice the recommended dose of a diet pill in order to avoid gaining weight after binge eating?** |  |  |  |  |
| No | 10 (100%) | 4 (100%) | 8 (100%) |  |
| Yes | 0 (0%) | 0 (0%) | 0 (0%) |  |
| Missing | 52 (83.9%) | 20 (83.3%) | 29 (78.4%) |  |
| **During the 3 months prior to coming to this program, have you withheld your use of insulin to try to control your weight?** |  |  |  |  |
| No | 8 (100%) | 4 (100%) | 2 (66.7%) | 0.2 |
| Yes | 0 (0%) | 0 (0%) | 1 (33.3%) |  |
| Missing | 54 (87.1%) | 20 (83.3%) | 34 (91.9%) |  |
| **During the 3 months prior to coming to this program, on average, how many hours per day did you spend watching TV, using a computer, and/or playing video games?** |  |  |  |  |
| None | 0 (0%) | 0 (0%) | 0 (0%) | 0.15 |
| 1 hour or less | 5 (8.3%) | 1 (4.2%) | 1 (2.9%) |  |
| 1-2 hours | 12 (20%) | 11 (45.8%) | 5 (14.3%) |  |
| 2-4 hours | 20 (33.3%) | 5 (20.8%) | 14 (40%) |  |
| More than 4 hours | 23 (38.3%) | 7 (29.2%) | 15 (42.9%) |  |
| Missing | 2 (3.2%) | 0 (0%) | 2 (5.4%) |  |
| **During the 3 months prior to coming to this program, how much of your daily food intake did you consume after suppertime?** |  |  |  |  |
| None | 23 (38.3%) | 5 (20.8%) | 11 (31.4%) | 0.59 |
| Up to a quarter | 26 (43.3%) | 13 (54.2%) | 17 (48.6%) |  |
| About half | 8 (13.3%) | 6 (25%) | 5 (14.3%) |  |
| More than half | 3 (5%) | 0 (0%) | 2 (5.71%) |  |
| Missing | 2 (3.2%) | 0 (0%) | 2 (5.4%) |  |
| **During the 3 months prior to coming to this program, how hungry were you on a usual morning?** |  |  |  |  |
| Not at all | 6 (10%) | 5 (20.8%) | 9 (25.7%) | 0.46 |
| A little | 24 (40%) | 9 (37.5%) | 12 (34.3%) |  |
| Somewhat | 16 (26.7%) | 5 (20.8%) | 8 (22.9%) |  |
| Moderately | 12 (20.0%) | 3 (12.5%) | 3 (8.6%) |  |
| Very | 2 (3.3%) | 2 (8.3%) | 3 (8.6%) |  |
| Missing | 2 (3.2%) | 0 (0%) | 2 (5.4%) |  |
| **During the 3 months prior to coming to this program, how often did you have trouble getting to sleep?** |  |  |  |  |
| Never | 14 (23.3%) | 7 (29.2%) | 10 (28.6%) | 0.04* |
| Sometimes | 23 (38.3%) | 11 (45.8%) | 8 (22.9%) |  |
| About half the time | 10 (16.7%) | 0 (0%) | 6 (17.1%) |  |
| Usually | 12 (20%) | 2 (8.3%) | 8 (22.9%) |  |
| Always | 1 (1.7%) | 4 (16.7%) | 3 (8.6%) |  |
| Missing | 2 (3.2%) | 0 (0%) | 2 (5.4%) |  |
| **Other than to use the bathroom, during the 3 months prior to coming to this program, how often did you get up at least once in the middle of the night?** |  |  |  |  |
| Never | 29 (48.3%) | 11 (45.8%) | 17 (48.6%) | 0.84 |
| Less than once a week | 8 (13.3%) | 5 (20.8%) | 7 (2%) |  |
| About once a week | 9 (1%) | 2 (8.3%) | 2 (5.71%) |  |
| More than once a week | 11 (18.3%) | 4 (16.7%) | 5 (14.3%) |  |
| Every night | 3 (5%) | 2 (8.3%) | 4 (11.4%) |  |
| Missing | 2 (3.2%) | 0 (0%) | 2 (5.4%) |  |
| **During the 3 months prior to coming to this program, when you got up in the middle of the night, how often did you snack?** |  |  |  |  |
| Never | 19 (61.3%) | 10 (76.9%) | 12 (70.6%) | 0.86 |
| Sometimes | 9 (2%) | 3 (23.1%) | 3 (17.6%) |  |
| About half the time | 0 (0%) | 0 (0%) | 1 (5.9%) |  |
| Usually | 2 (6.5%) | 0 (0%) | 1 (5.9%) |  |
| Always | 1 (3.2%) | 0 (0%) | 0 (0%) |  |
| Missing | 31 (50%) | 11 (45.8%) | 20 (54.1%) |  |
| **During the 3 months prior to coming to this program, were you in an occupation involving night or evening shifts or other unusual time requirements that interfere with meals?** |  |  |  |  |
| No | 51 (8%) | 21 (87.5%) | 31 (88.6%) | 0.94 |
| Yes | 9 (15%) | 3 (12.5%) | 4 (11.4%) |  |
| Missing | 2 (3.2%) | 0 (0%) | 2 (5.4%) |  |
| **During the 3 months prior to coming to this program, how often did you keep eating a meal even though you were not hungry anymore?** |  |  |  |  |
| Rarely or never | 15 (2%) | 9 (37.5%) | 13 (37.1%) | 0.5 |
| Occasionally (once per week) | 23 (38.3%) | 8 (33.3%) | 11 (31.4%) |  |
| Frequently (more than once per week) | 14 (23.3%) | 4 (16.7%) | 10 (28.6%) |  |
| Nearly every day | 8 (13.3%) | 3 (12.5%) | 1 (2.9%) |  |
| Missing | 2 (3.2%) | 0 (0%) | 2 (5.4%) |  |
| **During the 3 months prior to coming to this program, how often did you keep eating a meal even though you felt full?** |  |  |  |  |
| Rarely or never | 25 (41.7%) | 9 (37.5%) | 11 (31.4%) | 0.49 |
| Occasionally (once per week) | 21 (35.0%) | 6 (25%) | 17 (48.6%) |  |
| Frequently (more than once per week) | 10 (16.7%) | 6 (25%) | 6 (17.1%) |  |
| Nearly every day | 4 (6.7%) | 3 (12.5%) | 1 (2.9%) |  |
| Missing | 2 (3.2%) | 0 (0%) | 2 (5.4%) |  |
| **Do you currently smoke cigarettes?** |  |  |  |  |
| No | 60 (100%) | 24 (100%) | 35 (100%) |  |
| Yes | 0 (0%) | 0 (0%) | 0 (0%) |  |
| Missing | 2 (3.2%) | 0 (0%) | 2 (5.4%) |  |
| **Do you currently use other forms of tobacco, such as cigars, cigarillos, chewing tobacco, snuff, dip, etc.?** |  |  |  |  |
| No | 60 (100%) | 23 (95.8%) | 34 (97.1%) | 0.24 |
| Yes | 0 (0%) | 1 (4.2%) | 1 (2.9%) |  |
| Missing | 2 (3.2%) | 0 (0%) | 2 (5.4%) |  |
| **How often do you have a drink containing alcohol?** |  |  |  |  |
| Never | 53 (88.3%) | 22 (91.7%) | 31 (88.6%) | 0.71 |
| Monthly or less | 5 (8.3%) | 1 (4.2%) | 4 (11.4%) |  |
| 2-4 times per month | 2 (3.3%) | 1 (4.2%) | 0 (0%) |  |
| Missing | 2 (3.2%) | 0 (0%) | 2 (5.4%) |  |
| **How many drinks containing alcohol do you have on a typical day when you are drinking?** |  |  |  |  |
| 1-2 | 3 (42.9%) | 2 (100%) | 1 (25.0%) | 0.95 |
| 3-4 | 1 (14.3%) | 0 (0%) | 0 (0%) |  |
| 5-6 | 2 (28.6%) | 0 (0%) | 1 (25%) |  |
| 7-9 | 0 (0%) | 0 (0%) | 1 (25%) |  |
| 10 or more | 1 (14.3%) | 0 (0%) | 1 (25%) |  |
| Missing | 55 (88.7%) | 22 (91.7%) | 33 (89.2%) |  |
| **In the past 12 months, other than as prescribed by a physician, have you used any opiates?** |  |  |  |  |
| No | 60 (100%) | 24 (100%) | 35 (100%) |  |
| Yes | 0 (0%) | 0 (0%) | 0 (0%) |  |
| Missing | 2 (3.2%) | 0 (0%) | 2 (5.4%) |  |
| **In the past 12 months, other than as prescribed by a physician, have you used any amphetamines?** |  |  |  |  |
| No | 60 (100%) | 24 (100%) | 35 (100%) |  |
| Yes | 0 (0%) | 0 (0%) | 0 (0%) |  |
| Missing | 2 (3.2%) | 0 (0%) | 2 (5.4%) |  |
| **In the past 12 months, other than as prescribed by a physician, have you used any hallucinogens?** |  |  |  |  |
| No | 59 (98.3%) | 24 (100%) | 35 (100%) | 1 |
| Yes | 1 (1.7%) | 0 (0%) | 0 (0%) |  |
| Missing | 2 (3.2%) | 0 (0%) | 2 (5.4%) |  |
| **In the past 12 months, other than as prescribed by a physician, have you used any marijuana?** |  |  |  |  |
| No | 56 (93.3%) | 22 (91.7%) | 33 (94.3%) | 1 |
| Yes | 4 (6.7%) | 2 (8.3%) | 2 (5.7%) |  |
| Missing | 2 (3.2%) | 0 (0%) | 2 (5.4%) |  |
| **In the past 12 months, other than as prescribed by a physician, have you used any cocaine?** |  |  |  |  |
| No | 60 (100%) | 24 (100%) | 35 (100%) |  |
| Yes | 0 (0%) | 0 (0%) | 0 (0%) |  |
| Missing | 2 (3.2%) | 0 (0%) | 2 (5.4%) |  |
| **In the past 12 months, other than as prescribed by a physician, have you used any PCP/angel dust?** |  |  |  |  |
| No | 60 (100%) | 24 (100%) | 35 (100%) |  |
| Yes | 0 (0%) | 0 (0%) | 0 (0%) |  |
| Missing | 2 (3.2%) | 0 (0%) | 2 (5.4%) |  |
